# Smoking Ban and Small-For-Gestational Age Births in Ireland

**DOI:** 10.1371/journal.pone.0057441

**Published:** 2013-03-26

**Authors:** Zubair Kabir, Sean Daly, Vanessa Clarke, Sheila Keogan, Luke Clancy

**Affiliations:** 1 TobaccoFree Research Institute Ireland, Dublin, Ireland; 2 Department of Epidemiology & Public Health, University College Cork, Cork, Ireland; 3 Coombe Women and Infants University Hospital, Dublin, Ireland; The Ohio State Unversity, United States of America

## Abstract

**Background:**

Ireland introduced a comprehensive workplace smoke-free legislation in March, 2004. Smoking-related adverse birth outcomes have both health care and societal cost implications. The main aim of this study was to determine the impact of the Irish smoke-free legislation on small-for-gestationa- age (SGA) births.

**Methods and Findings:**

We developed a population-based birthweight (BW) percentile curve based on a recent study to compute SGA (BW <5^th^ percentile) and very SGA (vSGA - BW<3^rd^ percentile) for each gestational week. Monthly births born between January 1999 and December 2008 were analyzed linking with monthly maternal smoking rates from a large referral maternity university hospital. We ran individual control and CUSUM charts, with bootstrap simulations, to pinpoint the breakpoint for the impact of ban implementation ( = April 2004). Monthly SGA rates (%) before and after April 2004 was considered pre and post ban period births, respectively. Autocorrelation was tested using Durbin Watson (DW) statistic. Mixed models using a random intercept and a fixed effect were employed using SAS (v 9.2). A total of 588,997 singleton live-births born between January 1999 and December 2008 were analyzed. vSGA and SGA monthly rates declined from an average of 4.7% to 4.3% and from 6.9% to 6.6% before and after April 2004, respectively. No auto-correlation was detected (DW = ∼2). Adjusted mixed models indicated a significant decline in both vSGA and SGA rates immediately after the ban [(−5.3%; 95% CI −5.43% to −5.17%, p<0.0001) and (−0.45%; 95% CI: −0.7% to −0.19%, p<0.0007)], respectively. Significant gradual effects continued post the ban periods for vSGA and SGA rates, namely, −0.6% (p<0.0001) and −0.02% (p<0.0001), respectively.

**Conclusions:**

A significant reduction in small-for-gestational birth rates both immediately and sustained over the post-ban period, reinforces the mounting evidence of the positive health effect of a successful comprehensive smoke-free legislation in a vulnerable population group as pregnant women.

## Introduction

Secondhand smoke (SHS) exposure is a Group I carcinogen and there is no risk-free safe level of SHS exposure [1]. There is also substantial evidence that both direct (firsthand) and maternal exposure to SHS increase the risk of pregnancy complications [2], [3]. Maternal smoking during pregnancy is an important modifiable risk factor and has both immediate and long-term health consequences. For instance, mothers who smoked during pregnancy have a two-fold increased risk of having low birthweight (LBW) babies compared to non-smoking mothers [4], [5]. Many pregnant women continue to smoke, for example, one in five pregnant women continues smoking in Ireland [6].

The population health impact of comprehensive smoke-free policies is increasingly contributing to changing social norms of a society. However, the effect of anti-smoking policies on pregnant women is also of considerable interest both from a population health and an economic perspective. To date, three studies have documented that smoke-free legislations did have a positive impact both on maternal smoking rates and on some adverse birth outcomes, mainly preterm births and small-for-gestational age births [6]–[8]. Each study has its own methodological limitations and some strengths. However, it is imperative that similar studies are performed across different population settings to reinforce such growing evidence, which is limited at present.

Earlier we reported that maternal smoking rates in Ireland fell by 12% one year after the Irish smoke-free legislation was introduced in March 2004 [6]. The same study also reported a 25% reduction in overall preterm birth risks after the smoke-free legislation. A recent study in Scotland using retrospective cohort design added further evidence suggesting that small-for-gestational age (SGA) birth rates declined at least by 4.5% after the Scottish smoke-free legislation of 2006 [7]. The present study builds on the earlier Irish study and the most recent Scottish study to examine a possible impact of the Irish smoke-free legislation on one of the several smoking-related adverse birth outcomes, namely, SGA birth rates over a 10-year period (January 1999 and December 2008) pre-post the ban, employing retrospective secondary analyses of individual-level data. Unlike the Scottish study [7], the present study explores the effect both on late-stage of pregnancy (measured by the month of delivery) and on early-stage of pregnancy (measured by the month of conception). In addition, the present study employs a newly developed population birthweight percentile reference curve for the general Irish population to estimate SGA, unlike the Scottish study [7].

## Methods

### Ethics Statement

The Coombe Women and Infants University Hospital Ethics Committee and the Dublin Institute of Technology (DIT) Ethics Committee approved the study.

The National Perinatal Reporting System (NPRS) routinely collects birth information from all maternity hospitals in Ireland. Details on the NPRS can be accessed through the website (http://www.esri.ie/health_information/nprs/). A concept proposal was submitted to the ESRI (Economic and Social Research Institute) in Ireland to get approval for accessing the dataset. Individual-level data were acquired from the NPRS from January 1999 to December 2008 (10 years) for all births in Ireland. Only singleton live births were included in this analysis (n = 588,997).

Ireland does not have a birthweight (BW) percentile reference curve. Therefore, a reference BW percentile for the Irish population was developed to estimate SGA births, as discussed below. No sample size estimation was performed, as the datasets included all births across the whole of Ireland.

### Calculation and Validation of SGA Estimates

SGA was estimated based on a recent Lancet study [9] (details in Appendix S1), which derived a global reference foetal-weight and BW percentile that is easily adapted to any local population. We calculated mean BW at 40 weeks using singleton live-births (n = ∼60,000) for year 2007 (mean BW = 3,619 g) and the standard deviation ( = 11.98%). Employing an easily adaptable previously programmed Excel-based software, weight percentiles for gestational age 24 weeks and beyond was calculated (Figure S1 and Table S1 in Appendix S2). BWs below the reference BW against 3^rd^ and 5^th^ percentile for each gestational week thus computed were considered very SGA (vSGA) and SGA for the Irish population, respectively, similar to a recent study [10]. Average monthly SGA percentages were thus calculated for all 120 months (January 1999-December 2008).

For validation of the newly developed BW percentile curve, we downloaded a previously validated tool- the GRAW (Gestation Related Average Weight) Centile Calculator from the following site: https://www.gestation.net/fetal_growth/graw/index.htm.

We adopted the same principle above of inputting the mean BW at 40 weeks gestation ( = 3,619 g) for the year 2007 onto the centile tool to plot another population-based BW percentile chart (Table S2 in Appendix S2) both for comparison and for validation. It is reassuring that 10^th^ and 90^th^ centile limits against each gestational week ≥24 weeks for both these charts are broadly similar (tables S1 &S2 in Appendix S2).

### Month of Conception

The exact date of conception could not be calculated in the present study, as the exact dates of births of babies were not available to the study for ethical reasons. However, a close approximation to the month of conception was feasible. We calculated the month of conception subtracting gestational age (in weeks) from the month of delivery for each singleton live-birth. Next, we computed monthly SGA and vSGA rates (in percentages) by month of conception. Because of fewer cases in the extremes of month of conception, we excluded those live-births (totalling <0.5%) of the total 588,997 singleton live-births, and thus restricted to the conception period between April 1998 and March 2008 (120 months in total). The monthly conception period ranged from a maximum of 10 months (∼40–41 gestational weeks) to a minimum of 6 months (∼24 gestational weeks) with ±2 weeks variations. A quick run of the frequency distribution of gestational period of the study sample also revealed that >55% of the study subjects fall within 40–41 weeks of gestation.

### Outcome Measures

In this study, we proposed to use two main outcome measures – first, monthly SGA and vSGA rates (%) by the month of birth (January 1999-December 2008), and the second was monthly SGA and vSGA rates (%) by the month of conception (April 1998–March 2008). However, detailed outputs of the analyses pertaining to all singleton live-births (n = 588,997) by month of delivery are shown. Gestational age is primarily based on an early ultrasound examination in Ireland (>95% of cases) [6].

### Maternal Smoking Rates

The NPRS does not collect routine information on maternal smoking rates. Corresponding monthly maternal smoking rates were acquired from a tertiary large referral maternity hospital in Dublin for the final model estimates. Detailed information on the sources of maternal smoking data is available in our previous publication [6]. In brief, mothers at the time of registration were interviewed on a host of lifestyle, maternal and clinical characteristics, including smoking status as a routine data collection procedure which is inputted on an electronic clinical maternity system, the Euroking K2 maternity system. Current smoking status was derived from the self-reported affirmative responses to two questions: i) “Did you ever smoked? Yes/No” and “Are you currently smoking? Yes/No”.

Smoking rates for full calendar years were available from 2000–2008. First, individual-level maternal smoking rates were computed for all singleton live-births from the Coombe Women and Infants University database from 2000–2008 (n = ∼60,000 singleton live births). Next, individual-level maternal smoking rates for each SGA and vSGA births, as defined based on the newly derived birthweight percentile curve, were estimated. Third, monthly average smoking rates separately for SGA and vSGA babies were computed for each calendar year (2000–2008). Finally, aggregate monthly maternal smoking rates thus computed from 2000 to 2008 were linked with the NPRS dataset as against each individual SGA and vSGA births by month of birth.

Because of the likelihood of non-comparability in methodologies of both the datasets, sub-group analyses by smoking status were not computed for the present study. 65% of the Coombe Women and Infants University database comprises births from Dublin, and not surprisingly administrative-specific overall maternal smoking rates for all 30 administrative areas were highly skewed (appendix S4). Therefore, computing administrative-specific maternal smoking rates by SGA and vSGA for all 30 areas by each month may potentially lead to reducing the validity and precision of such estimates. However, mixed modelling techniques [as employed in the present study] accounting for any underlying clustering effects within and across these 30 administrative regions should indirectly adjust for variations in maternal smoking rates within and across these regions. Nonetheless, maternal smoking rates are self-reported- so there is always a possibility of recall bias.

### Analyses

A total of 120 months were observed between January 1999 and December 2008. First, we ran several individual control charts, including CUSUM charts, separately for both observed monthly SGA and vSGA rates (in percentages) by *month of delivery* to pinpoint a possible breakpoint for the effect of the smoke-free legislation using Taylor’s powerful change-point analysis tool. Details of this tool are available on the website (http://www.variation.com/cpa/tech/changepoint.html). Confidence intervals at various levels were computed based on non-replacement bootstrap simulations of 1000 samples to pinpoint the breakpoint. Based on such computations, month ‘64’ corresponding to April 2004 was the breakpoint for both SGA and vSGA births when significant changes occurred. Details of such computations are explained in Appendix S3.

We ran similar individual control and CUSUM charts (Figures S3 and S4 in Appendix S3) for monthly SGA and vSGA rates (%) *by month of conception* (April 1998-March 2008) to pinpoint a possible breakpoint for the effect of the smoke-free legislation using the same change-point-analysis tool. Table S3 in Appendix S3, in line with the explanations provided in Appendix S3 for tables S1 and S2 indicate that point ‘64’ (here refers to month May 2003) is the first most significant change point for monthly vSGA rates by month of conception. On closer examination, point ‘64’ corresponds to a lag of 10 months duration from the month of the Irish smoking ban ( = end of March 2004). Therefore, the breakpoint identified earlier using individual month of delivery as the unit of analysis, namely, April 2004, is consistent with using individual month of conception as the unit of analysis. A similar observation was noted for SGA rates by month of conception in table S4 of Appendix S3. Taken together, April 2004 can be confidently assigned as the breakpoint for the impact of the ban implementation.

Thus, January 1999–April 2004 were considered pre-ban period births, while May 2004–December 2008 was the post-ban period in the present study.

### Statistical Modelling

To start with, auto-correlation was assessed testing Durbin-Watson statistic by modelling monthly aggregate-level data (Proc AUTOREG) for both vSGA and SGA births, as well as factoring three time-varying covariates into each model: overall secular trend, gradual post-intervention trend (slope), and the immediate intervention level (step change). No auto-correlation of first order was detected (DW = ∼2.0). The estimates (intercepts and beta coefficients) from these models were used to estimate expected monthly vSGA and SGA rates by month of delivery, and to compare expected monthly rates with observed monthly rates.

Preliminary analyses showed variations in SGA and vSGA monthly rates within 30 administrative areas of Ireland indicating a clustering effect. Therefore, individual-level data were modelled using both a random intercept and a fixed effect model assuming dependency within geographic locations (administrative areas). A random intercept for SGA and vSGA implies that there is an average SGA and vSGA rate in the population, but there is also variability between geographic locations and within each geographic location. All such analyses were performed using Proc MIXED of SAS v9.2.

The final mixed model estimates were adjusted for several socio-demographic and physiological characteristics available to the study [sex of the child, maternal age, maternal and paternal occupational status, marital status, antenatal care, parity, maternal smoking rates], and the clustering effect of maternal residence within the 30 administrative areas. Time was included into the model as a continuous variable from month 1 in January 1999 to month 120 in December 2008 to capture overall secular trends in both SGA and vSGA rates over time. An indicator variable was used to define the smoking ban, with a value of zero given to the months before ban implementation and a value of one given for the month after the ban was implemented (April, 2004) and all following months to estimate the post-intervention level (step change). Finally, values of one were given for the breakpoint ( = April 2004) – one month of ban implementation and the subsequent months up to 1, 3, 6, or 12 months post-ban with all other months before the breakpoint denoted by a value of zero for estimating the gradual post-intervention trend (slope). Following model convergence, the goodness-of-fit of models was assessed using the Akaike Information Criterion (AIC) – the smaller the AIC better the model. The immediate intervention level (step change) and the gradual post-intervention trend (slope) both in vSGA and SGA monthly rates were computed, including robust standard error estimates.

Furthermore, a similar statistical modelling was employed to examine the impact of the ban implementation on SGA and vSGA monthly rates (in percentages) by the month of conception (details not shown) – April 1998- March 2008 (coded as ‘1’ = April 1998 to ‘120’ = March 2008).

Finally, to delineate the effects of premature babies, all singleton live-births known to be born premature (defined as births <37 completed gestational weeks) were excluded (totalling ∼8%) to run a similar statistical modelling for adjusted estimates of immediate post-intervention level (step change) and a gradual post-intervention trend (slope) in both monthly SGA and vSGA rates.

## Results

**Figure 1:**
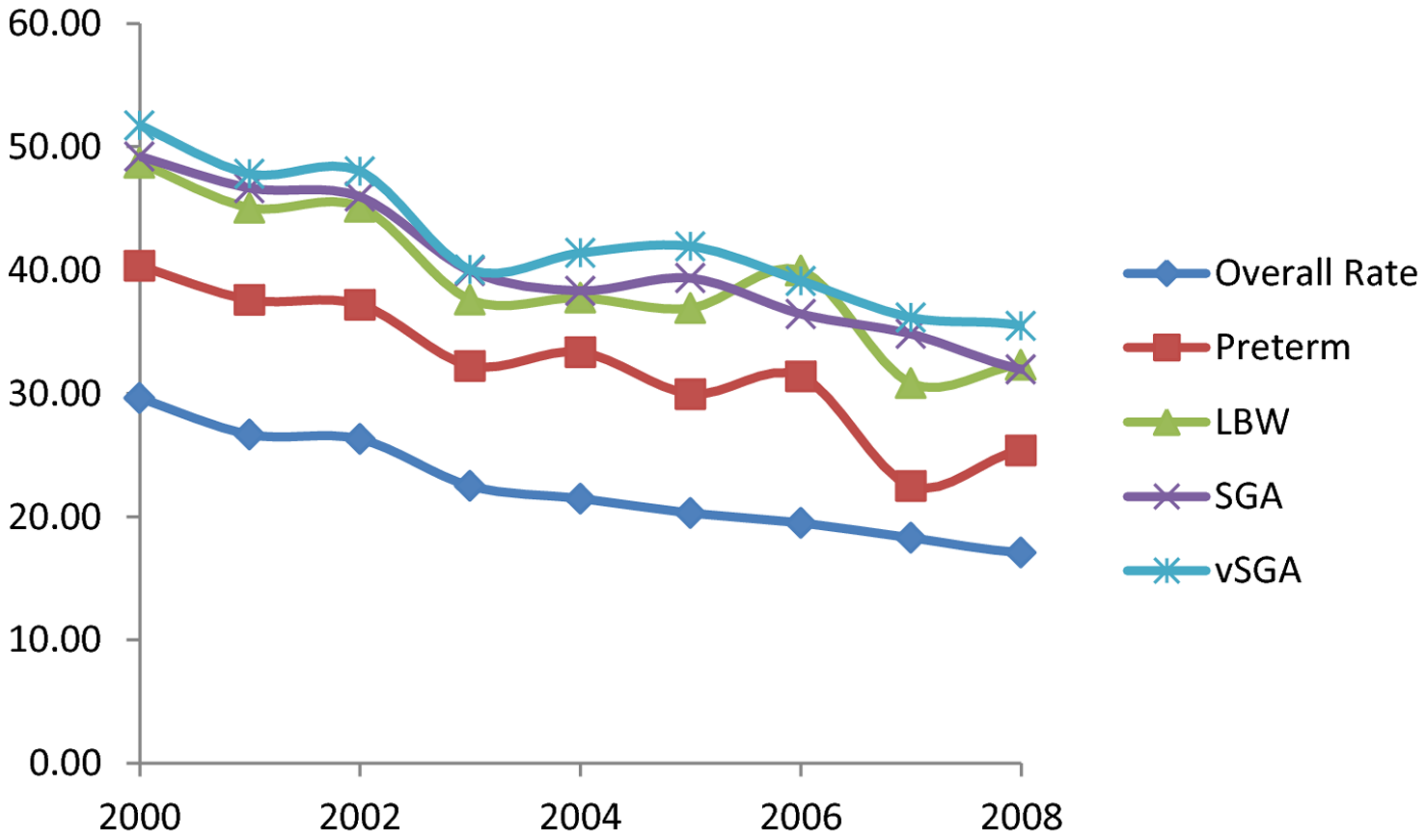
Yearly smoking rates (%) in pregnant women and in mothers who gave birth to low weight (LBW), preterm, small-for-gestational age (SGA) and very (vSGA) babies from 2000 to 2008.

Figure 1 shows yearly maternal smoking rates from 2000 to 2008 available from the Coombe Women and Infants University Hospital. Figure 1 also shows that yearly smoking rates among mothers who had SGA and vSGA births are relatively higher compared to yearly overall maternal rates, which remain high in Ireland.

**Figure 2:**
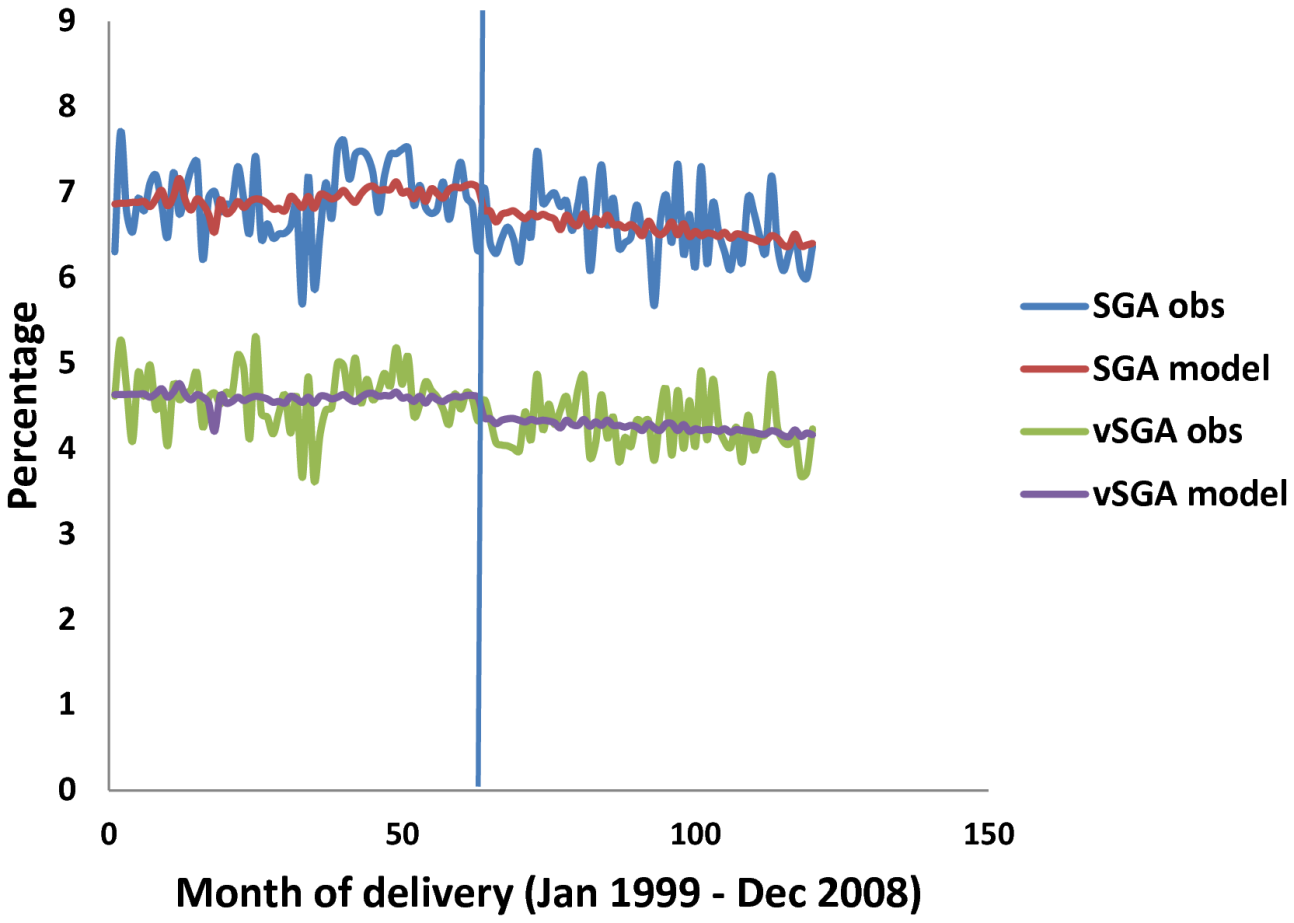
Monthly observed and modelled (expected) small-for-gestational age (SGA) and very SGA (vSGA) rates in Ireland by month of delivery, January 1999-December 2008 [the bar indicates month 64 ( = April 2004).

Figure 2 shows both the observed and the modelled (expected) estimates of vSGA and SGA monthly rates (in percentages) by month of delivery between January 1999 and December 2008 indicating a step change during month 64 corresponding to April 2004. The observed monthly vSGA rates before the ban were 4.7% on average which fell to an average of 4.3% in the post-ban period (row 64 in Table S1 of Appendix S3). A similar pattern was observed for SGA monthly rates (from an average of 6.9% to 6.6%, respectively, as shown in row 64 in Table S2 of Appendix S3).

Table 1 details the immediate post-intervention level (step change) and the gradual post-intervention trend (slope) estimates across different parameters of analyses following adjusted mixed modelling. The adjusted estimates indicate that vSGA rates showed a much greater immediate effect compared to SGA rates - a decline of −5.3% [95%CI: −5.43% to −5.17%; (p<0.0001)] compared to −0.45% [95%CI: −0.70% to −0.19%; (p = 0.0007)] decline in SGA rates. Gradual post-intervention effects in both vSGA and SGA rates were smaller- a monthly decline of −0.6% [SE 0.002; (p<0.0001)] and −0.02% [SE 0.004; (p<0.0001) in respective vSGA and SGA rates after the ban.

**Table 1:** Mixed model estimates* of immediate post-intervention level (step changes) and gradual post-intervention trends (slope) for monthly Small-for-gestational (SGA) and very SGA (vSGA) rates in Ireland, January 1999-December 2008.

Outcome measures	Immediate Effects (step change)		Gradual Effects (slope change)	
	Coefficients*100 [SE]	‘p’ value	Coefficients*100 [SE]	‘p’ value
**Month of delivery (n = 588,997)**				
vSGA	−5.3 [0.06]	<0.0001	−0.6 [0.002]	<0.0001
SGA	−0.45 [0.13]	0.0007	−0.02 [0.004]	<0.0001
**Excluding premature births (n = 541,039)**				
vSGA	−4.5 [0.064]	<0.0001	−0.58 [0.002]	<0.0001
SGA	−0.39 [0.13]	0.003	−0.018 [0.004]	<0.0001
**Excluding mat smoking (n = 588,997)				
vSGA	−2.7 [0.11]	0.01	−0.01 [0.003]	0.11
SGA	−0.31 [0.13]	0.02	−0.01 [0.004]	0.0005

* Adjusted for maternal smoking (not for **); maternal age; parity; sex of the baby; marital status; antenatal care; mother’s occupation; father’s occupation; secular trend, and clustering effect with and between 30 administrative regions.

SE = Standard Error.

Table 1 also shows that a significant −4.5% immediate post-intervention decline was observed in monthly vSGA rates when analyzed by the month of conception. In summary, immediate and gradual post-intervention effects were observed irrespective of modelling by the month of delivery or by the month of conception, and even excluding premature births.

## Discussion

This study quantifies the impact of a comprehensive smoke-free legislation on a potentially preventable adverse birth outcome, namely, small-for-gestational age births derived from a newly developed population-based birthweight percentile reference curve. The study findings indicate a significant decline of −5.3% in vSGA rates in Ireland immediately after the ban, which is in agreement with a recent Scottish study [7]. Significant gradual post-intervention trends were also observed, namely, a monthly decline of −0.6% in vSGA rates sustained throughout the post-ban periods, and of similar direction but of smaller magnitude for monthly SGA rates (−0.02%). Such observations add to the body of evidence indicating the positive health impact of comprehensive smoke-free legislations both in Ireland and in comparable populations elsewhere [12], [13]. The fact that greater effects were observed in vSGA babies than in SGA babies underscores both the physiological importance and the vulnerability of babies considered to be the ‘smallest’ biologically and also having the largest intra-uterine growth restriction in absolute terms. Furthermore, an estimated immediate post-intervention fall of −4.5% when analyzed by the month of conception indirectly suggests that the smoking ban had a similar effect during the early-stage of pregnancy.

Despite methodological challenges, the present study has several strengths. First, the development of an Irish population-based birthweight percentile reference curve for estimating SGA rates in Ireland is novel and timely. The Scottish study [7] despite the availability of a Scottish BW percentile reference curve [14] did not utilize such curves for SGA computations. Second, the present study validated the newly developed BW percentile curve using a previously validated GRAW (Gestation Related Average Weight) Centile Calculator [11]. Third, detailed analyses were performed to pinpoint and to delineate the most significant visible breakpoint rather than arbitrarily selecting the actual month of the Irish smoking ban implementation, utilizing a powerful change-point analysis tool across different units of analyses: individual month of births vs. individual month of conception. Fourth, employing a robust mixed modelling technique accounted for both clustering effects within and between geographic locations and the underlying secular trends before and after the smoke-free legislation, thus clearly taking advantage of individual-level data available for a longer period. Fifth, examining such effects both on early-stage of pregnancy (analysis by month of conception) and on late-stage of pregnancy (analysis by month of delivery), as well as exploring only ‘term’ singleton live-births - all providing similar estimates is reassuring. Finally, a large nationally representative sample of more than half a million singleton live-births clearly added statistical power to the study findings, thereby also avoiding selection bias.

The present study, however, has inherent methodological limitations. The cross-sectional retrospective study design limits causal inferences. Unmeasured/unidentified factors are more likely to introduce residual confounding. The linkage of maternal smoking rates available from a single large referral centre with a national perinatal reporting system data raises issues of external validity and generalization of maternal smoking rates. However, various sources of maternal smoking statistics in Ireland are consistent with the data utilized in this study [15]–[16]. In addition, the computations of monthly maternal smoking rates separately for SGA and vSGA births may have accounted in part for any underlying biases in population mean estimates, thus minimising ecologic fallacies. Although recall bias is a possibility using self-reported maternal smoking rates but evidence suggests that self-reported smoking habits (including SHS exposure) by pregnant women is in good agreement with objective measurements of cigarette exposure [17] or may be an underestimate [18].

The observed declines are biologically plausible. A recent Scottish study showed an immediate effect [7], more precisely, the same study showed an effect 3 months before the implementation of the Scottish ban. The present study captures both early and late effects of SHS exposure levels on pregnancy complications. A causal relationship is supported by a recent randomized intervention where infants born to mothers in the intervention group with reduced SHS exposure had a significant lower risk of adverse birth outcomes [19]. Active maternal smoking has detrimental effects on placental architecture, placental function, and early and late foetal growth, predisposing to a range of adverse birth outcomes [20]–[21]. Evidence also suggests that it is during the third trimester of pregnancy when smoking restricts foetal growth regardless of the previous smoking history [22]–[23]. Although difficult to delineate accurately the possible health effects across different periods of gestation, a similar positive health effect of the ban observed on SGA rates when analyzed by the month of conception would also indirectly capture any underlying effects on the early-stage of pregnancy. A recent study in the US also determined how a population-level intervention (here a smoke-free policy) could translate into improvements at the individual level through reductions in preterm birth risks [8]. Nevertheless, it is always difficult to extrapolate changes in smoking behaviour among the general population to pregnant women [24], but the marginal fall in maternal smoking behaviour that was observed earlier in Ireland is also plausible.^6^ In addition, completely smoke-free homes are increasing in Ireland [25]–[26]. No significant changing obstetric practices were reported in Ireland coinciding with the ban implementation. However, the general observations in recent years of increasing elective Caesarean rates or a shift to higher average age of pregnant women, as well as a rise in obese mothers giving births may have influenced the study findings but are more likely to bias toward the null [24].

In conclusion, positive health effects after the introduction of the Irish smoke-free legislation are mounting [12], [27]. The growing evidence in support of the positive population health gains of smoke-free policies for a vulnerable population, such as pregnant women on pregnancy complications, is both encouraging and crucial [24]. In addition to immediate gains, smoking-related adverse birth outcomes are preventable considering the long-term health care and societal cost implications. Future studies of similar nature should also address the methodological limitations akin to cross-sectional ecologic designs or retrospective secondary data analytical study designs. Details on personalized information on changing maternal obesity patterns or on maternal smoking exposure levels across different socio-economic groups, as well as changing obstetric practices may provide additional insights into future studies.

## Supporting Information

Appendix S1 The Lancet Study- supplementary material webappendix 1- Mikolajczyk RT, Zhang J, Betran AP, Souza JP, Mori R, Gulmezoqlu AM, Merialdi M. A global reference for fetal-weight and birthweight percentiles. Lancet 2011; 377: 1855–61.
(DOCX)

Appendix S2 Figure S1, Birthweight percentile reference curve for the Irish population (based on the Lancet study). Table S1, Birthweight percentile reference values for the Irish population (based on the Lancet study). Table S2, Birthweight percentile reference values for the Irish population (based on the GRAW tool).
(DOCX)

Appendix S3 Figure S1, A control chart showing changes to monthly vSGA rates (by month of birth) in the background using change-point analyses tool outputs. Figure S2, A CUSUM chart showing changes to monthly vSGA rates (by month of birth) in the background using change-point analyses tool outputs. Figure S3, A control chart showing changes to monthly vSGA rates (by month of conception) in the background using change-point analyses tool outputs. Figure S4, A CUSUM chart showing changes to monthly vSGA rates (by month of conception) in the background using change-point analyses tool outputs. Table S1, Significant detection points and bootstrap simulations for monthy vSGA rates (by month of birth) using change-point analyses tool outputs. Table S2, Significant detection points and bootstrap simulations for monthy SGA rates (by month of birth) using change-point analyses tool outputs. Table S3, Significant detection points and bootstrap simulations for monthy vSGA rates (by month of conception) using change-point analyses tool outputs. Table S4, Significant detection points and bootstrap simulations for monthy SGA rates (by month of conception) using change-point analyses tool outputs.
(DOCX)

Appendix S4 Overall maternal smoking rates (%) for singleton live-births (n = ∼60,000) in the Coombe Women and Infants University Hospital, Dublin between 2000 and 2008 across 30 administrative areas in the Republic of Ireland.
(DOCX)
