# Supplementary material for: Smoking Ban and Small-For-Gestational Age Births in Ireland
Source: PLoS One. 2013 Mar 26;8(3):e57441. doi: 10.1371/journal.pone.0057441 (PMC3608631; doi:10.1371/journal.pone.0057441)
Supplement: Appendix S1 — The Lancet Study- supplementary material webappendix 1- Mikolajczyk RT, Zhang J, Betran AP, Souza JP, Mori R, Gulmezoqlu AM, Merialdi M. A global reference for fetal-weight and birthweight percentiles. Lancet 2011; 377: 1855–61. (DOCX) [file pone.0057441.s001.docx]

**APPENDIX SI (The Lancet Study- supplementary material webappendix 1)***

***** Mikolajczyk RT, Zhang J, Betran AP, Souza JP, Mori R, Gulmezoqlu AM, Merialdi M. A global

reference for fetal-weight and birthweight percentiles. Lancet 2011; 377: 1855-61.
