# Supplementary material for: Smoking Ban and Small-For-Gestational Age Births in Ireland
Source: PLoS One. 2013 Mar 26;8(3):e57441. doi: 10.1371/journal.pone.0057441 (PMC3608631; doi:10.1371/journal.pone.0057441)
Supplement: Appendix S2 — Figure S1, Birthweight percentile reference curve for the Irish population (based on the Lancet study). Table S1, Birthweight percentile reference values for the Irish population (based on the Lancet study). Table S2, Birthweight percentile reference values for the Irish population (based on the GRAW tool). (DOCX) [file pone.0057441.s002.docx]

**APPENDIX SII**

**Figure S1: Birthweight percentile reference curve for the Irish population (based on the Lancet study)**

**Table S1: Birthweight percentile reference values for the Irish population (based on the Lancet study)**

| **Gestational age** |  |  |  |  |  | **Percentile** |  |  |  |  |  |
| --- | --- | --- | --- | --- | --- | --- | --- | --- | --- | --- | --- |
|  | **99th** | **97th** | **95th** | **90th** | **75th** | **mean** | **25th** | **10th** | **5th** | **3rd** | **1st** |
| **24** | 902 | 865 | 845 | 815 | 765 | 709 | 653 | 603 | 572 | 553 | 516 |
| **25** | 1053 | 1010 | 987 | 952 | 893 | 828 | 762 | 704 | 668 | 646 | 602 |
| **26** | 1221 | 1171 | 1144 | 1104 | 1035 | 960 | 884 | 816 | 775 | 748 | 698 |
| **27** | 1405 | 1348 | 1317 | 1270 | 1192 | 1105 | 1018 | 939 | 892 | 862 | 804 |
| **28** | 1607 | 1541 | 1506 | 1452 | 1363 | 1263 | 1163 | 1074 | 1020 | 985 | 919 |
| **29** | 1824 | 1749 | 1709 | 1648 | 1547 | 1433 | 1320 | 1219 | 1158 | 1118 | 1043 |
| **30** | 2055 | 1971 | 1926 | 1858 | 1743 | 1616 | 1488 | 1373 | 1305 | 1260 | 1176 |
| **31** | 2300 | 2206 | 2156 | 2079 | 1951 | 1808 | 1665 | 1537 | 1460 | 1410 | 1316 |
| **32** | 2556 | 2451 | 2396 | 2310 | 2168 | 2009 | 1850 | 1708 | 1622 | 1567 | 1462 |
| **33** | 2820 | 2704 | 2643 | 2549 | 2392 | 2217 | 2042 | 1884 | 1790 | 1729 | 1613 |
| **34** | 3089 | 2963 | 2896 | 2793 | 2620 | 2429 | 2237 | 2064 | 1961 | 1894 | 1768 |
| **35** | 3361 | 3223 | 3150 | 3038 | 2850 | 2642 | 2433 | 2246 | 2133 | 2061 | 1923 |
| **36** | 3630 | 3482 | 3403 | 3281 | 3079 | 2854 | 2628 | 2426 | 2305 | 2226 | 2077 |
| **37** | 3894 | 3734 | 3650 | 3520 | 3302 | 3061 | 2819 | 2602 | 2472 | 2387 | 2228 |
| **38** | 4147 | 3977 | 3887 | 3748 | 3517 | 3260 | 3002 | 2771 | 2632 | 2542 | 2372 |
| **39** | 4385 | 4205 | 4110 | 3964 | 3719 | 3447 | 3175 | 2930 | 2784 | 2688 | 2509 |
| **40** | 4604 | 4416 | 4316 | 4162 | 3905 | 3619 | 3334 | 3077 | 2923 | 2823 | 2634 |
| **41** | 4800 | 4604 | 4500 | 4339 | 4071 | 3773 | 3476 | 3208 | 3047 | 2943 | 2746 |

**Table S2**: **Birthweight percentile reference values for the Irish population (based on the GRAW tool)**
