# Supplementary material for: Smoking Ban and Small-For-Gestational Age Births in Ireland
Source: PLoS One. 2013 Mar 26;8(3):e57441. doi: 10.1371/journal.pone.0057441 (PMC3608631; doi:10.1371/journal.pone.0057441)
Supplement: Appendix S3 — Figure S1, A control chart showing changes to monthly vSGA rates (by month of birth) in the background using change-point analyses tool outputs. Figure S2, A CUSUM chart showing changes to monthly vSGA rates (by month of birth) in the background using change-point analyses tool outputs. Figure S3, A control chart showing changes to monthly vSGA rates (by month of conception) in the background using change-point analyses tool outputs. Figure S4, A CUSUM chart showing changes to monthly vSGA rates (by month of conception) in the background using change-point analyses tool outputs. Table S1, Significant detection points and bootstrap simulations for monthy vSGA rates (by month of birth) using change-point analyses tool outputs. Table S2, Significant detection points and bootstrap simulations for monthy SGA rates (by month of birth) using change-point analyses tool outputs. Table S3, Significant detection points and bootstrap simulations for monthy vSGA rates (by month of conception) using change-point analyses tool outputs. Table S4, Significant detection points and bootstrap simulations for monthy SGA rates (by month of conception) using change-point analyses tool outputs. (DOCX) [file pone.0057441.s003.docx]

**APPENDIX SIII**

In brief, table S1 in Appendix SIII detects two significant changes for vSGA rates - first change at point 40 and the second one at point 64. However, on closer examination additional information suggests that 95% CI is wider for the first change at point 40 compared to that at point 64. In addition, point 64 showed significant changes with 100% confidence. Therefore, point 64 is more likely to be the period when significant changes did occur for vSGA births. Likewise, table S2 in Appendix III detects several significant changes for SGA rates. However, point 64 is again showing a significant change with 99% confidence, and more importantly, point 64 is a level 1 change suggesting that point 64 is the first significant change to be detected and is more visible. Taken together, it becomes clear that the change-point analyses for both the outcomes studied did detect significant changes just prior to point 64, which corresponds to April 2004 - a month following the actual introduction of the Irish smoke-free legislation, namely, March 2004. Thus, SGA and vSGA births before month 64) were considered pre-ban period (January 1999-April 2004) births and those after month 64 were post-ban period (May 2004-December 2008) births in the present study.

**Figure S1: A control chart showing changes to monthly vSGA rates (by month of birth) in the background using change-point analyses tool outputs**

**Figure S2: A CUSUM chart showing changes to monthly vSGA rates (by month of birth) in the background using change-point analyses tool outputs**

**Figure S3: A control chart showing changes to monthly vSGA rates (by month of conception) in the background using change-point analyses tool outputs**

**Figure S4: A CUSUM chart showing changes to monthly vSGA rates (by month of conception) in the background using change-point analyses tool outputs**

**Table S1: Significant detection points and bootstrap simulations for monthy vSGA rates (by month of birth) using change-point analyses tool outputs**

**Table S2: Significant detection points and bootstrap simulations for monthy SGA rates (by month of birth) using change-point analyses tool outputs**

**Table S3: Significant detection points and bootstrap simulations for monthy vSGA rates (by month of conception) using change-point analyses tool outputs**

**Table S4: Significant detection points and bootstrap simulations for monthy SGA rates (by month of conception) using change-point analyses tool outputs**
