# Supplementary material for: Smoking Ban and Small-For-Gestational Age Births in Ireland
Source: PLoS One. 2013 Mar 26;8(3):e57441. doi: 10.1371/journal.pone.0057441 (PMC3608631; doi:10.1371/journal.pone.0057441)
Supplement: Appendix S4 — Overall maternal smoking rates (%) for singleton live-births (n = ∼60,000) in the Coombe Women and Infants University Hospital, Dublin between 2000 and 2008 across 30 administrative areas in the Republic of Ireland. (DOCX) [file pone.0057441.s004.docx]

**APPENDIX SIV**

**Overall maternal smoking rates (%) for singleton live-births (n=~60,000) in the Coombe Women and Infants University Hospital, Dublin between 2000 and 2008 across 30 administrative areas in the Republic of Ireland**

| **Regions** | **Rates (%)** |
| --- | --- |
| Armagh | 0 |
| Carlow | 16.9 |
| Cavan | 9.8 |
| Clare | 0 |
| Cork | 23.5 |
| Dublin | 24.9 |
| Down | 0 |
| Fermanagh | 0 |
| Galway | 14.7 |
| Kerry | 20 |
| Kildare | 15.8 |
| Kilkenny | 7.5 |
| Laois | 17.8 |
| Leitrim | 12.7 |
| Limerick | 18.2 |
| Longford | 15.2 |
| Louth | 12.1 |
| Mayo | 7.1 |
| Meath | 14.3 |
| Monaghan | 3.9 |
| Offaly | 14.1 |
| Roscommon | 9.4 |
| Sligo | 3.2 |
| Tipperary | 8.5 |
| Tyrone | 100 |
| Waterford | 25 |
| Westmeath | 13.9 |
| Wexford | 13.4 |
| Wicklow | 18.5 |
